# Generation of V-point polarization singularity using single phase encoding with a spatial light modulator

**DOI:** 10.1038/s41598-022-27337-x

**Published:** 2023-01-06

**Authors:** Praveen Kumar, A. Srinivasa Rao, Takashige Omatsu

**Affiliations:** 1 Graduate School of Science and Engineering, Chiba University, Chiba, 263-8522 Japan; 2 Molecular Chirality Research Center, Chiba University, Chiba, 263-8522 Japan

**Keywords:** Optics and photonics, Physics

## Abstract

A liquid crystal Spatial Light Modulator (SLM) can be used in various ways to produce vector-vortices. Superposition of scalar vortices with orthogonal polarization is a common approach, while a more recent technique is to use dual-phase modulation. These approaches require modulation of at least two phase patterns with a SLM or multiple SLMs. In this paper, we propose a novel technique to produce vector-vortices by modulating orthogonal light components through a single phase pattern with a SLM. It does not require interferometric setups, and simplifies the generation of light beams with V-point polarization singularities. Because of compact and robustness of our experimental setup, it can be easily integrated to any device for applications of vector-vortices.

## Introduction

The importance of optical vortices has grown significantly in recent years due to their unique properties, which have enabled a plethora of applications^1,2^. Vortex beams are characterized by a helical-shaped wavefront which creates a phase singularity along their beam axis, resulting in a doughnut-shaped intensity profile. As-such, they are often referred to as doughnut beams. The optical field of a vortex beam can be expressed as,

1 $$E_{VB} = u(x,y)\exp \left( {i\ell \tan^{ - 1} \left( {{y \mathord{\left/ {\vphantom {y x}} \right. \kern-0pt} x}} \right)} \right),$$

where *x* and *y* are transverse coordinates. The amplitude distribution is given by *u*(*x*,*y*) and *ℓ* is the azimuthal index or the topological charge (TC). Optical fields can possess a polarization singularity, which can be classified as C-points or V-points^3,4^. C-point singularities can be found in Poincaré beams, while cylindrical-vector beams hold V-points along their beam axis^5^. They have a spatially non-uniform polarization distribution across the transverse plane and have unique propagation properties arising from Gouy phase effects^6,7^. Poincaré beams have zero TC in one of their orthogonal components. They are particular case of hybrid vector beams which holds different value of the TC on the orthogonal components. On the other hand, the TC on the orthogonal components in cylindrical-vector beams have equal magnitude but opposite sign. They can be represented as paraxial solutions of the vector Helmholtz equation and poses an advantage in a wider range of applications^8^. They are also referred to as vector-vortex (VV) beams.

The VV beams of different types and with different orders can be represented on a high-order Poincaré sphere^9^. Commonly known radially and azimuthally polarized VV beams exhibit unusual properties when focused. For example, when focused, a radially polarized beam creates a longitudinal electric field component at the focal point along the optical axis^10^. This feature is useful in applications such as optical super-resolution microscopy. Furthermore, higher order VV beams can have controlled field distributions in their focused core which may be useful in applications such as optical trapping and laser machining^11,12^.

Generally, the VV beams can be produced in a number of ways, these include their direct generation from a laser cavity, or via the conversion of a laser beam using external-cavity elements such as diffractive optical elements^8,13,14^. Programmable electro-optic devices such as Digital Micromirror Devices (DMD) and Liquid Crystal Spatial Light Modulators (LC SLM) have emerged as powerful tools for the generation of VV beams^15–18^. VV beams consisting of V-points can be produced through the superposition of two orthogonally polarized vortex beams with an equal magnitude but opposite sign of TC^19^. Various interferometric arrangements such as Mach Zehnder or Sagnac interferometers have been used for the superposition of beams^2,5^. This approach offers flexibility in producing VV beams but the method is highly sensitive to external vibrations. Other techniques include the use of q-plates, and S-wave-plates^20–22^. They are particular cases of geometric phase elements and can be fabricated with liquid–crystal technology or as metasurfaces. These are widely employed techniques which are commercially available. Their main advantage relies on the compactness of the optical setups and simplicity of use, although not being reconfigurable. The drawback of these approaches is that they are designed for specific VV beams. Several attempts have been made to find a solution which combines both flexibility and a simple implementation. Emphasis was made to simplify the process using techniques that do not involve interferometric arrangements for the superposition of scalar beam components^23–26^. Single SLM-based techniques have also been proposed to reduce cost^27–30^. In this approach, only half the area of the SLM's display is available for modulation of each beam components, and therefore a SLM with high-spatial resolution and optimized phase response is required^31,32^. As such, it is clear that there remains significant scope for the development of techniques for the efficient generation of VV beams.

In this paper, we demonstrate a technique to convert laser beams into VV beams through the modulation of a single-phase pattern with a LC SLM and without any requirement of additional interferometric arrangements for beam superposition. The implementation of this approach relies on dual-pass phase modulation and does not require any interferometric arrangements thus simplifying alignment. The proposed scheme paves the way for rapid and straightforward generation of VV beams with V-point singularities for commercial applications. Since the proposed method is based on modulating orthogonal light components with a single phase pattern, it cannot directly generate arbitrary vector beams or hybrid vector beams, such as the Poincaré beams.

## Principle

**Figure 1:**
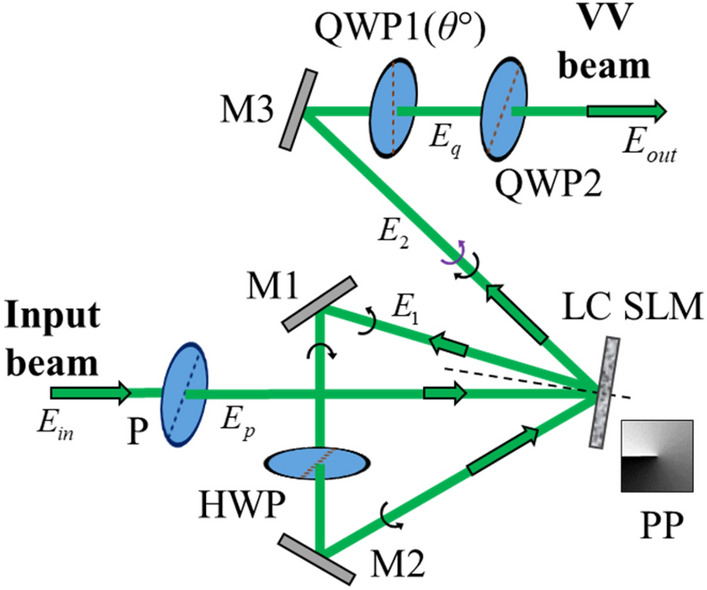
Schematic diagram showing the experimental setup used for vector beam generation. *P* polarizer, *M* mirror, *HWP* half-wave plate, *QWP* quarter-wave plate, *PP* phase pattern encoded on SLM.

The proposed technique for VV beam generation is described using Jones matrix formalism and the schematic diagram of the experimental setup is shown in Fig. 1.

The complex field amplitude of the input beam in the transverse *x*–*y* plane is denoted as *E*_*in*_(*x,y*). It is assumed to be a Gaussian beam with spatially uniform polarization distribution^6^. The input beam is transmitted through a linear polarizer with its polarization axis at 45° to the *x*-axis. The Jones vector of the resultant beam, *E*_*p*_(*x,y*) is expressed as,

2 $$E_{p} = \frac{{E_{in} }}{\sqrt 2 }\;\left[ {\begin{array}{*{20}c} 1 \\ 1 \\ \end{array} } \right].$$

The wavefront of the beam is controlled by a reflective-type LC SLM through phase modulation. If the slow axis of the SLM is fixed along the *x*-axis, the phase in the *x*-component of the beam is modulated while the *y*-component of the beam remains unmodulated. Utilizing this feature, the optical set-up is designed to control the phase of both components of light independently though two modulations. In the first modulation, the SLM encodes the phase distribution *β*(*x,y*) along the *x*-component. The Jones vector of the resultant beam is obtained by multiplication of the Jones matrix of the SLM with the incident beam as follows,

3 $$E_{1} = \left[ {\begin{array}{*{20}c} {e^{ - i\beta (x,y)} } & 0 \\ 0 & 1 \\ \end{array} } \right]\,E_{p} = \frac{{E_{in} }}{\sqrt 2 }\left[ {\begin{array}{*{20}c} {e^{ - i\beta (x,y)} } \\ 1 \\ \end{array} } \right],$$

where *E*_1_(*x,y*) represents the beam after the first reflection from the SLM. *β*(*x,y*) represents the azimuthally-varying phase distribution over the range 0 to 2π. It is evaluated as,

4 $$\beta (x,y) = \ell \tan^{ - 1} \left( {{y \mathord{\left/ {\vphantom {y x}} \right. \kern-0pt} x}} \right).\,sign\left[ {L_{p}^{\left| \ell \right|} \left( {\frac{{2\left( {x^{2} + y^{2} } \right)}}{{w_{ \circ }^{2} }}} \right)} \right],$$

where ‘*sign*’ denotes the Signum function. The associated Laguerre polynomial is denoted as $L_{p}^{\left| \ell \right|}$ with *ℓ* being the azimuthal index and *p* is the radial index. By using reflecting mirrors M1, and M2, the modulated light is directed again to the same SLM for a second reflection. Before the reflection, the beam is passed through a half-wave plate (HWP) to rotate the polarization by 90° as the LC SLM modulates only the light component parallel to its slow axis. The axis of the HWP is kept at 45° to the *x*-axis. The change in polarization of light induced by the HWP is represented as follows,

5 $$E_{h} = \left[ {\begin{array}{*{20}c} 0 & 1 \\ 1 & 0 \\ \end{array} } \right]\,E_{1}^{{}} = \frac{{E_{in} }}{\sqrt 2 }\left[ {\begin{array}{*{20}c} 1 \\ {e^{ - i\beta } } \\ \end{array} } \right].$$

The term *E*_*h*_(*x,y*) represents the Jones vector of the modulated beam after the HWP. Modulation of the spatially varying phase distribution *β*(*x,y*) makes the wavefront helical. Using mirrors, the beam is directed again to the SLM for another cycle of modulation. The direction of the wavefront helicity becomes inverted due to reflection (by two mirrors and the SLM), and this is denoted by the opposite sign of TC. Mathematically, the term *E*_h_*(*x,y*) is used to denote the complex conjugate of *E*_*h*_(*x,y*), which has an opposite sign of TC. In the second modulation cycle, the phase distribution *β*(*x,y*) is again encoded on the reflected beam but now along the other component. The Jones vector of the resultant beam, denoted as *E*_2_(*x,y*) is expressed as,

6 $$E_{2} = \left[ {\begin{array}{*{20}c} {e^{ - i\beta } } & 0 \\ 0 & 1 \\ \end{array} } \right]\,{E_{h}}^{*} = \frac{{E_{in} }}{\sqrt 2 }\left[ {\begin{array}{*{20}c} {e^{ - i\beta } } \\ {e^{i\beta } } \\ \end{array} } \right].$$

The beam is then transmitted through a quarter wave plate (QWP) to introduce a phase shift between its orthogonal components. The angle between the *x*-axis and the slow axis of the QWP is denoted as *θ*. QWP1 introduces a phase delay in different components of light for *θ* = 90° and 0°, respectively. Both of these cases are represented as follows,

7 $$E_{{q(\theta = 90^{ \circ } )}} = \left[ {\begin{array}{*{20}c} { - i} & 0 \\ 0 & 1 \\ \end{array} } \right]\,E_{2} = \frac{{E_{in} }}{\sqrt 2 }\left[ {\begin{array}{*{20}c} { - ie^{ - i\beta } } \\ {e^{i\beta } } \\ \end{array} } \right],$$

8 $$E_{{q(\theta = 0^{ \circ } )}} = \left[ {\begin{array}{*{20}c} 1 & 0 \\ 0 & { - i} \\ \end{array} } \right]\,E_{2} = \frac{{E_{in} }}{\sqrt 2 }\left[ {\begin{array}{*{20}c} {e^{ - i\beta } } \\ { - ie^{i\beta } } \\ \end{array} } \right].$$

The term *E*_*q*_(*x,y*) represents the Jones vector of the resultant optical field. The beam is finally transmitted through a second QWP with its slow axis along 45° with the *x*-axis to change the polarization basis from linear to circular. The output beam *E*_*out*_(*x,y*) is expressed as follows,

9 $$E_{out} = \frac{1}{2}\,\left[ {\begin{array}{*{20}c} {1 - i} & {1 + i} \\ {1 + i} & {1 - i} \\ \end{array} } \right]\;E_{{q(\theta = 90^{ \circ } ,{\kern 1pt} 0^{ \circ } )}} .$$

The above equation represents beams that have vortices of order − *ℓ* and + *ℓ* in their orthogonal components in the circular basis of polarization. The resultant light field due to its propagation within the set-up after phase modulation is evaluated using the Fresnel diffraction formula. Different orders of VV beam are represented by different TC values of the vortices carried by the beam.

## Experimental system

**Figure 2:**
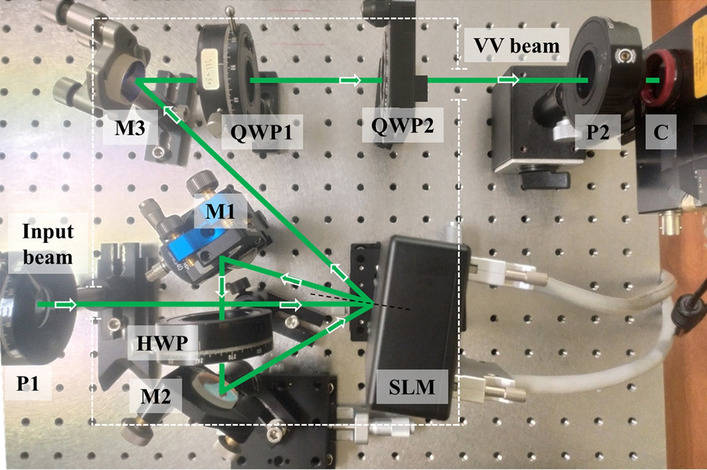
Photograph of the experimental set-up used for VV beam generation with the beam path overlaid in green and components labelled-*P* polarizer, *M* mirror, *HWP* half-wave plate, *QWP* quarter-wave plate, *C* camera.

Our experiment was performed with the aim of generating VV beams such as a radially and azimuthally polarized beams. The experimental set-up used is shown in Fig. 2. A collimated laser beam with a Gaussian profile is used as the input beam. It has wavelength of 532 nm and a spatially uniform polarization. The incident beam diameter on the SLM was maintained to ~ 6 mm. The beam was transmitted through a linear polarizer (P1) to ensure the beam was polarized at 45° to the *x*-axis (horizontal plane of the optical table). The beam was projected onto a reflective-type SLM (X10468-04, Hamamatsu Japan) which had a resolution of 792 × 600 pixel and pixel pitch of 20 µm. Its wavelength operation range is 510 ± 50 nm. This SLM was sensitive to light polarized along its slow axis that lies along *x*-axis. The phase pattern was encoded into the SLM as an 8-bit image. It had gray levels varying from 0 to 255, corresponding to phase delays of the light across the range 0 to 2π.

After modulation by the SLM, the beam acquired a phase of *exp*(*− iβ*) in one of its components. To modulate the other component of the beam via a second modulation cycle by the SLM, the beam was first transmitted through a HWP that interchanged its vertical and horizontal components. Two reflecting mirrors (M1 and M2) were used to redirect the beam towards the SLM. These mirrors may have induced small (but negligible in this experiment) depolarization effects. After the second modulation, the horizontal component acquired the new phase *exp*(*− iβ*) whereas the phase of the vertical component became *exp*(+ *iβ*) because of wavefront helicity inversion due to additional reflections. To control the relative phase between the beam components, the beam was transmitted through QWP1 whose slow axis could be rotated to 0° or 90° from the *x*-axis. The VV beam was finally obtained after the polarization basis conversion performed by QWP2 having its slow axis at 45° to the *x*-axis. The mirror M3 is not essential part of the system and has been used to direct the beam towards camera (CCD sensor, BeamGage Standard). Camera has resolution of 1928 × 1448 pixel and 3.69 µm pixel pitch.

The approach described herein enables phase control of beam components on a one-by-one basis as the beam propagates through the setup. Hence, this approach does not require additional interferometric arrangements for superposition of beams. Also, the same phase pattern displayed in the SLM is used for modulation of both components. These features greatly simplify the method of generating VV beams.

## Results and analysis

**Figure 3:**
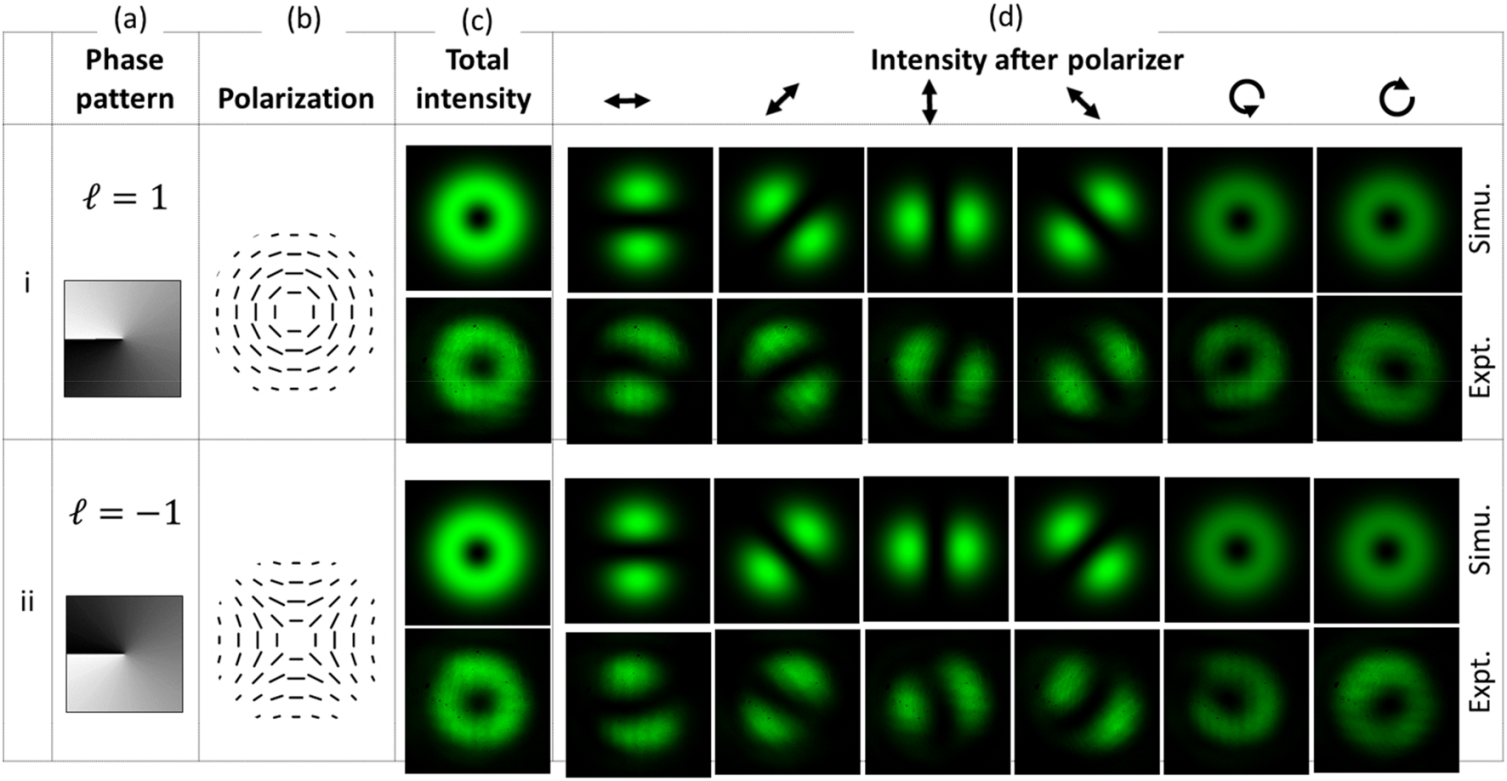
Simulation and experimental results showing the generation of VV beams for *θ* = 90° where rows (i) and (ii) corresponds to Type I and II beams, respectively.

Generic V-point polarization singularities include those designated as Type I, II, III and IV^31^. VV fields encompassing these types have been generated in this work. In order to generate Type I and II beams, the slow axis of the QWP1 was kept along the *y*-axis, such that *θ* = 90°. In this configuration, the Jones vector of the resultant optical field is given by Eq. (9) which is simplified as follows,

10 $$E_{{out(\theta = 90^{ \circ } )}} = \left[ {\begin{array}{*{20}c} {(1 + i)( - e^{ - i\beta } + e^{i\beta } )} \\ {(1 - i)(e^{ - i\beta } + e^{i\beta } )} \\ \end{array} } \right]\,\;\frac{{E_{in} }}{2\sqrt 2 },$$

where *E*_*in*_ represents the incident beam with a planar wavefront. Results showing the generated Type I and II beam are presented in rows (i) and (ii) of Fig. 3, respectively. Both beams required modulation of the azimuthally varying phase but with opposite sign of TC. The phase patterns encoded on the SLM required for the generation of each beam type are shown in column (a) of Fig. 3. These patterns were obtained from Eq. (4) while also considering the TC value of *ℓ* = 1 for Type I and *ℓ* = − 1 for Type II beams. The radial index remained zero. Columns (b) and (c) of Fig. 3 show the polarization and the intensity distributions of the beam. The intensity distribution of the beams under propagation was evaluated using the Fresnel diffraction formula^3^. Their polarization distribution was verified by observing the change in intensity distribution caused after transmitting the beams through a linear polarizer. Column (d) shows the spatial intensity profile of the beams at different transmission angles of the polarizer. Orientation angles 0°, 90°, 45°, 135° from the horizontal axis correspond to horizontal, vertical, + 45° and − 45° linear polarization components. Right- and left-handed circular polarization components are also recorded while inserting a QWP before the polarizer at transmission angles 45° and 135°. These intensity recordings allow measurement of Stokes parameters for the analysis of polarization distribution (refer to supplementary file).

The slow axis of QWP1 was changed along the *x*-axis for generation of Type III and IV beams, such that *θ* = 0°. In this case, the Jones vector of the resultant beam represented by Eq. (9) is simplified as,

11 $$E_{{out(\theta = 0^{ \circ } )}} = \left[ {\begin{array}{*{20}c} {(1 - i)(e^{ - i\beta } + e^{i\beta } )} \\ {(1 + i)(e^{ - i\beta } - e^{i\beta } )} \\ \end{array} } \right]\,\;\frac{{E_{in} }}{2\sqrt 2 }.$$

**Figure 4:**
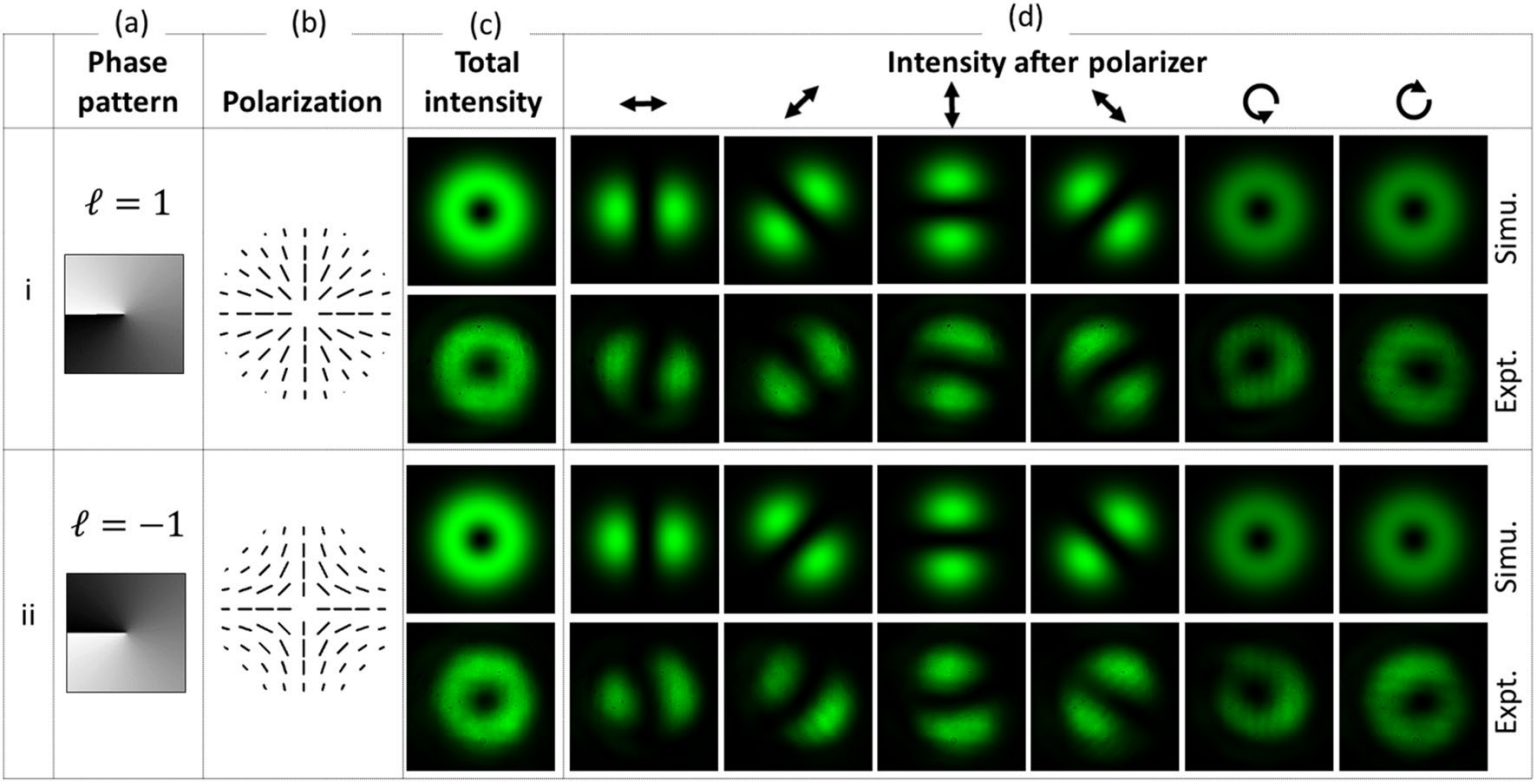
Simulation and experimental results showing the generation of VV beams for *θ* = 0°, where rows (i) and (ii) corresponds to Type III and IV beams, respectively.

The above equation represents Type III and IV beams when considering the TC as *ℓ* = 1 and − 1, respectively in the expression of *β* in Eq. (4). The experimental results detailing the generation of these types of beam are summarized in Fig. 4. The phase pattern encoded on the SLM for the generation of the beam types are shown in column (a), while column (b) and (c) show the polarization and spatial intensity profiles of the beams respectively. Column (d) shows the change in beam intensity as a result of varying the transmission angle of the polarizer, which verifies the polarization distribution of beam. The Poincaré–Hopf (PH) index describes the polarization distribution around a V-point^5^. In the case of Type I and III beams, the PH index is + 1, while for Type II and IV beams it is − 1.

**Figure 5:**
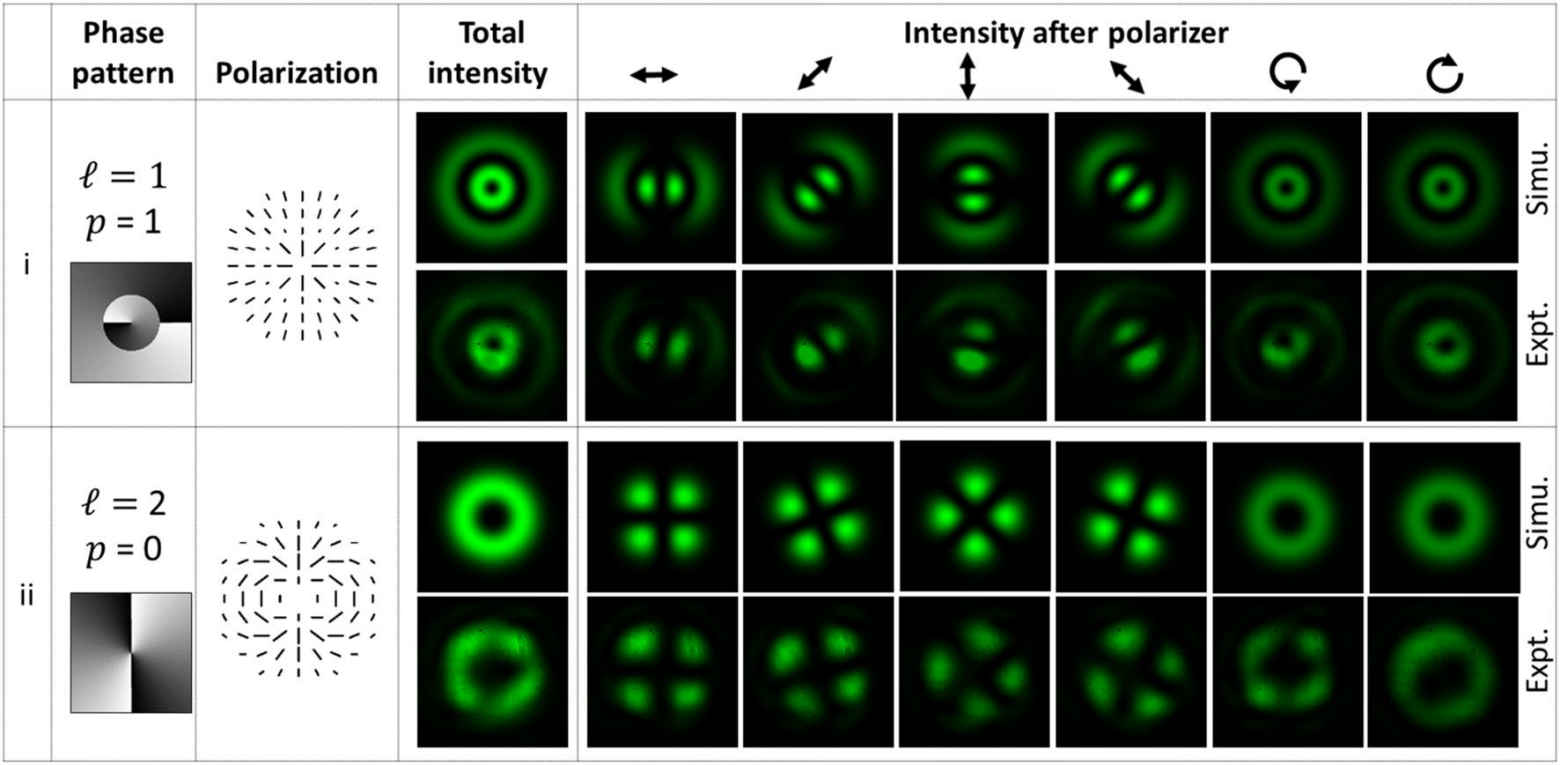
Simulation and experimental results for generation of higher order VV beams. Row (i) corresponds to a beam with non-zero radial index and row (ii) shows a beam of higher TC value.

High-order VV beams were also generated as a means of demonstrating the flexibility of the developed technique. To generate a VV beam with non-zero radial index, the required phase pattern was obtained through solution of Eq. (4) with for *ℓ* = 1 and *p* = 1. Figure 5(i) shows the encoded phase pattern, polarization and recorded intensity of the generated beam. Similarly, the phase pattern for *ℓ* = 2 was also determined and encoded onto the SLM. The results summarizing the properties of this beam are shown in Fig. 5(ii). These results demonstrate the successful generation of high-order VV beams. Other types vector fields and other orders of VV beams can be also generated using the appropriate phase pattern. Analysis of experimental results and simulation shown in Figs. 3, 4 and 5 is performed using MATLAB software^33^.

The correlation coefficient (CC) between the experimental and simulated total intensity of the generated beams is calculated for comparison. The CC value close to 1 indicates very close agreement between the images. The CC value for Type I, II, III, and IV beams shown in Figs. 3 and 4 are 0.9557, 0.9147, 0.9712 and 0.9525, respectively. CC value is also calculated for high-order beams shown in Fig. 5. It is 0.7130 for the case *ℓ* = 1, *p* = 1 while for *ℓ* = 2, *p* = 0, the CC value is 0.8728. It shows close agreement between the experimental and simulation results. The misalignment in the optical setup, phase response errors of SLM, and the deformation of SLM’s backplane could be the possible origin for slight asymmetry in the experimental intensity distribution of beams. In the present experiment, a correction mask for the deformation of SLM’s backplane and its spatially varying phase response error has not been applied. Hence there is scope to improve the beam quality further.

Previous works have also demonstrated the generation of vector beams using single SLM^5,16^. However, most of the papers use two phase patterns to modulate orthogonal components of the beam while using half of SLM’s display for each^32^. Moreover, various methods also use interferometric arrangements for beam superposition which may have alignment issues^15,19^. In contrast, the present approach uses a single-phase pattern and a non-interference-based technique, hence simplifying the generation of vector-vortices. However, the present method cannot directly generate arbitrary vector beams or hybrid vector beams.

## Conclusion

In conclusion, a technique to produce VV beams from the encoding of a single phase pattern through a LC SLM has been demonstrated. Unlike conventional approaches based on the superposition of scalar beams, the present technique does not require the use of any interferometric setups. Another advantage is the use of a single phase pattern encoded on the SLM to generate VV beams. This technique offers a simple approach for the modulation of orthogonal polarization basis of laser beams. Furthermore, the use of non-interferometric arrangements in the set-up simplifies the optical alignment and may provide robustness against external vibrations. This method is suitable for rapid generation of VV beams with V-point singularity of different orders. We anticipate that this robust method for vector field generation can be easily utilized for commercial applications.

## Supplementary Information

Supplementary Information.

## Data Availability

Data underlying the results presented in this paper are not publicly available at this time but may be obtained from the corresponding author (Praveen Kumar) upon reasonable request.
