# Supplementary material for: Generation of V-point polarization singularity using single phase encoding with a spatial light modulator
Source: Sci Rep. 2023 Jan 6;13:315. doi: 10.1038/s41598-022-27337-x (PMC9822894; doi:10.1038/s41598-022-27337-x)
Supplement: Supplementary file 1 — Supplementary Information. [file 41598_2022_27337_MOESM1_ESM.docx]

**Generation of V-point polarization singularity using single phase encoding with a spatial light modulator**

Praveen Kumar^1,2,*^, A Srinivasa Rao^1,2^ and Takashige Omatsu^1,2^

^1^Graduate School of Science and Engineering, Chiba University, Chiba, 263-8522, Japan

^2^Molecular Chirality Research Center, Chiba University, Chiba, 263-8522, Japan

^*^Corresponding author: praveenkumar6394@gmail.com

**Supplementary information**

**1. Jones matrix of wave plate**

Present work uses wave plates in the experimental set-up to generate vector beams. The general expression of the Jones matrix of a wave plate is described as,

$\left[\begin{matrix} \cos(-\theta) & \sin(-\theta) \\ -\sin(-\theta) & \cos(-\theta) \end{matrix}\right] \left[\begin{matrix} 1 & 0 \\ 0 & e^{-i\phi} \end{matrix}\right] \left[\begin{matrix} \cos\theta& \sin\theta\\ -\sin\theta& \cos\theta\end{matrix}\right]$ (S1)

where *φ* is the phase delay to be introduced between the orthogonal components of incident light and *θ* is the angle between slow axis of wave plate and the *x*-axis (laboratory horizontal).

Jones matrix of a half-wave plate (HWP) having slow axis at 45° from the *x*-axis is obtained by substituting, *φ = π* and *θ = π/4* is expressed as,$\left[\begin{matrix} 0 & 1 \\ 1 & 0 \end{matrix}\right]$.

Similarly, Jones matrix of a quarter-wave plate (QWP) having slow axis at 0° from the *x*-axis is obtained by substituting, *φ = π/2* and *θ = 0*  is expressed as, $\left[\begin{matrix} 1 & 0 \\ 0 & -i \end{matrix}\right]$.

Jones matrix of a QWP having slow axis at 45° from the *x*-axis is obtained by substituting, *φ = π/2* and *θ = π/4* is expressed as, $\frac{1}{2} \left[\begin{matrix} 1-i & 1+i \\ 1+i & 1-i \end{matrix}\right]$.

Jones matrix of a QWP having slow axis at 90° from the *x*-axis is obtained by substituting, *φ = π/2* and *θ = π/2* is expressed as, $\left[\begin{matrix} -i & 0 \\ 0 & 1 \end{matrix}\right]$.

**2. Derivation of Equation 10**

Equation 9 represents the general expression of vector-vortex (VV) beams that can be generated using the proposed method. This equation can represent beams of Type I and II while considering slow axis of QWP1 along *y*-axis. Hence, Eq. 10 is derived while considering *θ* = 90° in Eq. 9 as follows,

$E_{out}=\frac{1}{2} \left[\begin{matrix} 1-i & 1+i \\ 1+i & 1-i \end{matrix}\right] E_{q(\theta=90^{\circ})}$ (S2a)

By substituting the expression of $E_{q(\theta=90^{\circ})}$from Eq. 7, we get

$E_{out(\theta=90^{\circ})}=\frac{1}{2} \left[\begin{matrix} 1-i & 1+i \\ 1+i & 1-i \end{matrix}\right] \left[\begin{matrix} -ie^{-i\beta} \\ e^{i\beta} \end{matrix}\right] \frac{E_{in}}{\sqrt{2}}$ (S2b)

$E_{out(\theta=90^{\circ})}=\left[\begin{matrix} -ie^{-i\beta}(1-i)+e^{i\beta}(1+i) \\ -ie^{-i\beta}(1+i)+e^{i\beta}(1-i) \end{matrix}\right] \frac{E_{in}}{2\sqrt{2}}$ (S2c)

$E_{out(\theta=90^{\circ})}=\left[\begin{matrix} -e^{-i\beta}(1+i)+e^{i\beta}(1+i) \\ e^{-i\beta}(1-i)+e^{i\beta}(1-i) \end{matrix}\right] \frac{E_{in}}{2\sqrt{2}}$ (S2d)

$E_{out(\theta=90^{\circ})}=\left[\begin{matrix} (1+i)(-e^{-i\beta}+e^{i\beta}) \\ (1-i)(e^{-i\beta}+e^{i\beta}) \end{matrix}\right] \frac{E_{in}}{2\sqrt{2}}$ (S2e)

**3. Derivation of Equation 11**

Equation 9 can represent VV beams of Type III and IV while considering slow axis of QWP1 along *x*-axis. Hence, Eq. 11 is derived while considering *θ* = 0° in Eq. 9 as follows,

$E_{out}=\frac{1}{2} \left[\begin{matrix} 1-i & 1+i \\ 1+i & 1-i \end{matrix}\right] E_{q(\theta=0^{\circ})}$ (S3a)

By substituting the expression of $E_{q(\theta=0^{\circ})}$from Eq. 8, we get

$E_{out(\theta=0^{\circ})}=\frac{1}{2} \left[\begin{matrix} 1-i & 1+i \\ 1+i & 1-i \end{matrix}\right] \left[\begin{matrix} e^{-i\beta} \\ -ie^{i\beta} \end{matrix}\right] \frac{E_{in}}{\sqrt{2}}$ (S3b)

$E_{out(\theta=0^{\circ})}=\left[\begin{matrix} e^{-i\beta}(1-i)-ie^{i\beta}(1+i) \\ e^{-i\beta}(1+i)-ie^{i\beta}(1-i) \end{matrix}\right] \frac{E_{in}}{2\sqrt{2}}$ (S3c)

$E_{out(\theta=0^{\circ})}=\left[\begin{matrix} e^{-i\beta}(1-i)+e^{i\beta}(1-i) \\ e^{-i\beta}(1+i)-e^{i\beta}(1+i) \end{matrix}\right] \frac{E_{in}}{2\sqrt{2}}$ (S3d)

$E_{out(\theta=0^{\circ})}=\left[\begin{matrix} (1-i)(e^{-i\beta}+e^{i\beta}) \\ (1+i)(e^{-i\beta}-e^{i\beta}) \end{matrix}\right] \frac{E_{in}}{2\sqrt{2}}$ (S3e)

**4. Analysis of polarization states of the VV beams**

Equation 9 represents the resultant VV beams as $E_{out}(x,y)$. Its transverse components can be represented as $E_{x}(x,y)$ and $E_{y}(x,y)$. In terms of Jones vectors, it can be written as,

$E_{out}(x,y)=\left[\begin{matrix} E_{x}(x,y) \\ E_{y}(x,y) \end{matrix}\right]$ (S4a)

The spatial distribution of polarization states of the VV beams can be described using Stokes polarimetry. The transverse components given by the above equation$E_{x}(x,y)$ and $E_{y}(x,y)$are related to Stokes parameters as,

$\begin{matrix} S_{0}=\left\langle E_{x}E_{x}^{*}\right\rangle+\left\langle E_{y}E_{y}^{*}\right\rangle\\ S_{1}=\left\langle E_{x}E_{x}^{*}\right\rangle-\left\langle E_{y}E_{y}^{*}\right\rangle\\ S_{2}=\left\langle E_{x}E_{y}^{*}\right\rangle+\left\langle E_{y}E_{x}^{*}\right\rangle\\ S_{3}=i\left\langle E_{x}E_{y}^{*}\right\rangle-i\left\langle E_{y}E_{x}^{*}\right\rangle\end{matrix}$ (S4b)

where $\left\langle\ldots\right\rangle$ denotes the time average. The measurement of Stokes parameters is done by passing the beam through a QWP and a linear polarizer at different sets of retardation $\phi$ and polarizer transmission angle $\varphi$. The intensity can be expressed in terms of Stokes parameters as follows,

$I(\phi,\varphi)=0.5\left(S_{0}+S_{1}\cos2\varphi+S_{2}\cos\phi\sin2\varphi+S_{3}\sin\phi\sin2\varphi\right)$ (S4c)

Stokes parameters are obtained using the above relation from the following intensity measurements *I*(0°, 0°), *I*(0°, 45°), *I*(0°, 90°) *I*(0°, 135°), *I*(90°, 45°), and *I*(90°, 135°). The polarization distribution across the transverse plane of light beams are obtained by evaluating the polarization ellipse at different spatial locations. The polarization ellipse is expressed in terms of two angular parameters, which are related to Stokes parameter as,

$\begin{matrix} \tan\left(2\psi\right)=S_{2}/S_{1} \\ \sin\left(2\chi\right)=S_{3}/S_{0} \end{matrix}$ (S4d)

The azimuth angle, $\psi$ gives the orientation angle of the major axis of polarization ellipse from a reference axis (say *x*-axis) and lies within$[-\pi/2,\pi/2]$. The ellipticity angle $\chi$ decides the handedness of the polarization ellipse and lies in the range $[-\pi/4,\pi/4]$. The positive and negative ellipticity angle correspond to left and right handed polarization ellipse, respectively. For example, the ellipticity angle, $\chi=-\pi/4$ and $\chi=\pi/4$ correspond to right and left circular polarization, respectively. This description is useful to characterize the VV beams embedded with polarization singularities.

Figure 3 shows the recorded intensity of generated VV beams of Type I and II while Fig. 4 shows the results of Type III and IV. The azimuth angle $\psi(x,y)$ and ellipticity angle $\chi(x,y)$distributions are obtained for these beams which are shown in Fig. S1. Columns (a-d) corresponds to Type I, II, III, and IV, respectively. Rows (i) and (ii) presents the azimuth and ellipticity angle, respectively. They are obtained from the recorded beam intensity shown in Figs. 3 and 4. Corresponding simulation results are also presents as insets for comparison. It shows very close agreement between the experimental results and simulation. Row (iii) present the experimentally measured polarization distribution of the beam across the transverse plane. These results confirm the generation of a V-point singularity.


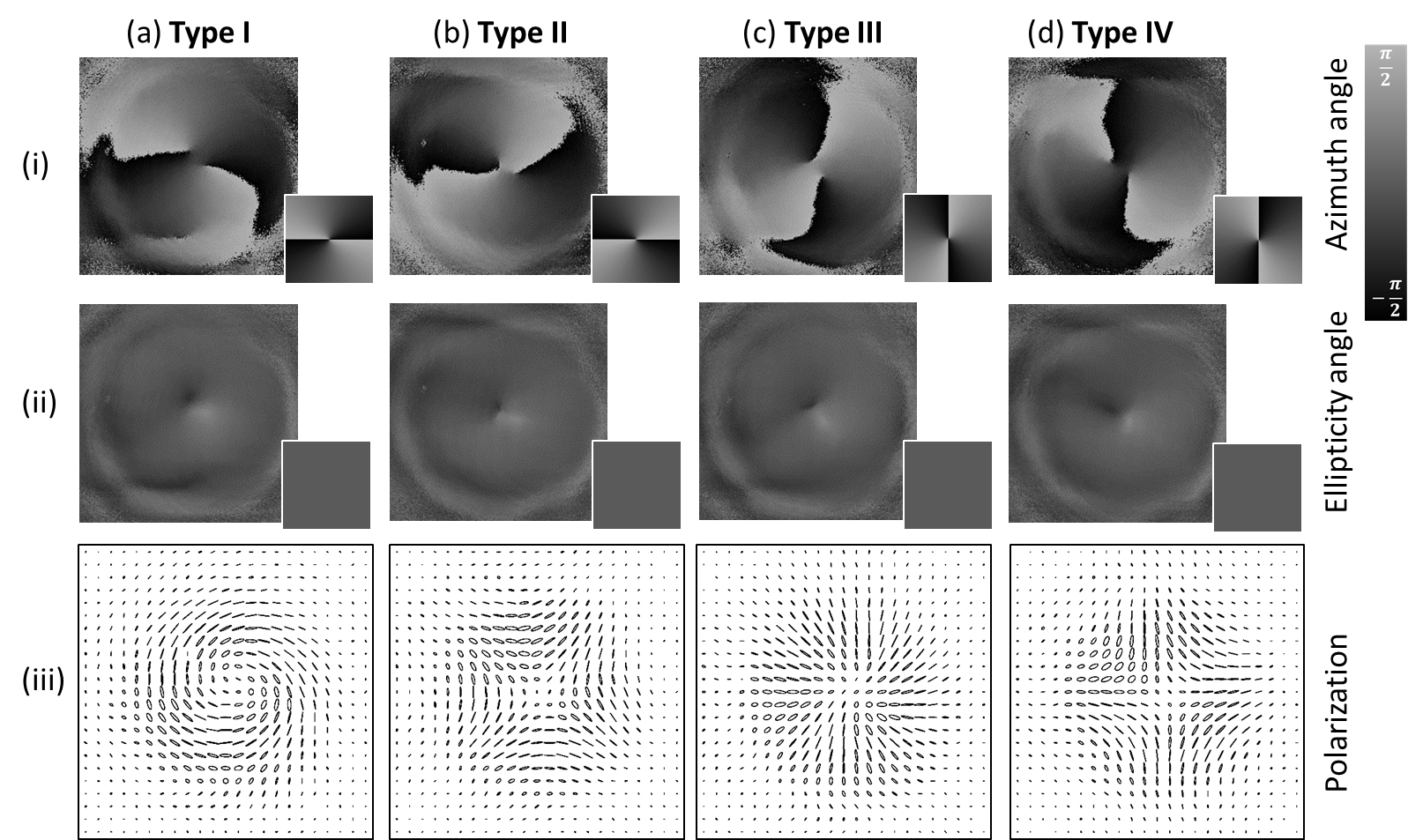


**Figure S1.** Analysis of polarization states of the VV beams of Type I, II, III, and IV, which are shown in Fig. 3 and 4. Rows (i) and (ii) presents the azimuth $\psi(x,y)$ and ellipticity angle $\chi(x,y)$ distributions, respectively. Row (iii) present the polarization distribution.

**5. Generation of beam with spiral-shaped polarization distribution**

The present set-up can also generate VV beams with spiral-shaped polarization distribution by removing the QWP1 from the optical set-up, which is presented in Figs. 1 and 2. In this case, the expression of output beam is represented as follows,

$E_{out}=\frac{1}{2} \left[\begin{matrix} 1-i & 1+i \\ 1+i & 1-i \end{matrix}\right] E_{2}$ (S5a)

By substituting the expression of $E_{2}$from Eq. 6, we get following equation that represents beam with spiral polarization distribution,

$E_{out}=\left[\begin{matrix} e^{-i\beta}(1-i)+e^{i\beta}(1+i) \\ e^{-i\beta}(1+i)+e^{i\beta}(1-i) \end{matrix}\right] \frac{E_{in}}{2\sqrt{2}}$ (S5b)

Results of the VV beams generated by removing the QWP1 from the set-up are presented in Fig. S2. Generation of this beam also requires modulation of azimuthally varying phase distribution using the spatial light modulator. The phase pattern encoded is obtained from Eq. 4 while considering the topological charge value as *ℓ* = 1. The radial index remains zero. The encoded phase pattern, polarization and the intensity distributions of the beam are shown in different columns of Fig. S2. The intensity profile of the beam as it transmits through a linear polarizer at different transmission angles is recorded. These results verify the beam's polarization distribution. The recorded intensity distributions of the beam are also in good agreement with the corresponding simulation results that confirm the generation of the VV beam with spiral-shaped polarization distribution.


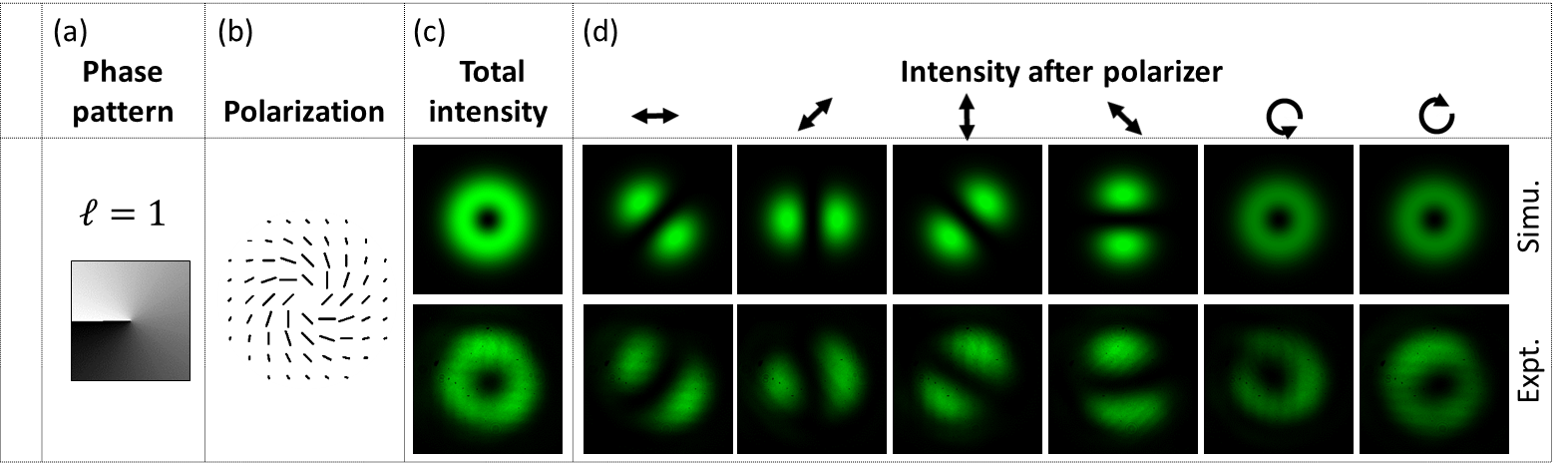


**Figure S2.** Results of generated VV beam with spiral-shaped polarization distribution.
